# Caregiver burden by treatment and clinical characteristics of patients with glioblastoma

**DOI:** 10.1007/s00520-021-06514-0

**Published:** 2021-09-11

**Authors:** Trang H. Au, Connor Willis, Maija Reblin, Katherine B. Peters, Phioanh Leia Nghiemphu, Jennie W. Taylor, Howard Colman, Adam L. Cohen, D. Ryan Ormond, Arnab Chakravarti, Nicole Willmarth, Jyothi Menon, Junjie Ma, Hillevi Bauer, Alexandre H. Watanabe, Cornelia M. Ulrich, Prianka Singh, Alexander Marshall, Beata Korytowsky, David Stenehjem, Diana Brixner

**Affiliations:** 1 Department of Pharmacotherapy, College of Pharmacy, University of Utah, Salt Lake City, UT USA; 2 Department of Health Outcomes and Behavior, Moffitt Cancer Center, Tampa, FL USA; 3 The Preston Robert Tisch Brain Tumor Center, Duke University Medical Center, Durham, NC USA; 4 Department of Neurology, University of California, Los Angeles, Los Angeles, CA USA; 5 Departments of Neurology and Neurological Surgery, University of California, San Francisco, San Francisco, CA USA; 6 Department of Neurosurgery, Huntsman Cancer Institute, University of Utah, Salt Lake City, UT USA; 7 Division of Oncology, Department of Internal Medicine, Huntsman Cancer Institute, University of Utah, Salt Lake City, UT USA; 8 Department of Neurosurgery, University of Colorado School of Medicine, Aurora, CO USA; 9 Department of Radiation Oncology, The Ohio State University College of Medicine, Columbus, OH USA; 10 American Brain Tumor Association, Chicago, IL USA; 11 Huntsman Cancer Institute and Department of Population Health Sciences, University of Utah, Salt Lake City, UT USA; 12 Bristol Myers Squibb, Princeton, NJ USA; 13 Department of Pharmacy Practice and Pharmaceutical Sciences, College of Pharmacy, University of Minnesota, Duluth, MN USA

**Keywords:** Glioblastoma, GBM, Caregiver, Cognitive dysfunction

## Abstract

**Background:**

Glioblastoma is an incurable disease with a poor prognosis. For caregivers of people with glioblastoma, the burden of care can be high. Patients often present with different clinical characteristics, which may impact caregiver burden in different ways. This study aimed to evaluate associations between patient clinical characteristics and caregiver burden/quality of life (QoL).

**Methods:**

Caregiver–patient dyads were enrolled at 7 academic cancer centers in the United States. Eligible caregiver participants were self-reported as the primary caregiver of an adult living with glioblastoma and completed a caregiver burden survey. Eligible patients were age ≥ 18 years at glioblastoma diagnosis and alive when their respective caregiver entered the study, with the presence of cognitive dysfunction confirmed by the caregiver. Data were analyzed with descriptive statistics and multivariable analyses.

**Results:**

The final cohort included 167 dyads. Poor patient performance status resulted in patient difficulty with mental tasks, more caregiving tasks, and increased caregiving time. Language problems were reported in patients with left-sided lesions. Patient confusion was negatively associated with all caregiver domains: emotional health, social health, general health, ability to work, confidence in finances, and overall QoL. Better caregiver QoL was observed in patients with frontal lobe lesions versus non-frontal lobe lesions.

**Conclusion:**

This study reinforced that patient performance status is a critical clinical factor that significantly affects caregiver burden, caregiving tasks, and caregiver time. Additionally, patient confusion affects multiple facets of caregiver burden/QoL. These results could be used to support guided intervention for caregiver support, customized to the patient experience.

**Supplementary Information:**

The online version contains supplementary material available at 10.1007/s00520-021-06514-0.

## Introduction

Glioblastoma is the most aggressive central nervous system malignancy [36]. The disease is associated with a median overall survival of 15 months and a 5-year survival rate of < 5% [36]. Glioblastoma is incurable, so the treatment goal is typically preserving quality of life (QoL). Standard of care for newly diagnosed glioblastoma is surgery followed by combination radiation and temozolomide (TMZ) therapy [12, 26]. Most patients develop recurrence 6 to 9 months after primary treatment [20]. Factors for more favorable prognosis include O^6^-methylguanine-DNA methyltransferase promoter (MGMT) methylation and the presence of isocitrate dehydrogenase (*IDH1*) mutation, whereas increased age, poor patient performance status, increased number of lesions, and subtotal resection of tumor at surgery (compared with gross total resection) are associated with poor prognosis [8, 29, 32, 37, 40]. Cognitive dysfunction may be present at the time of glioblastoma diagnosis and generally worsens over time due to disease sequelae and/or treatment toxicity [17].

Poor prognosis, aggressive disease course, and cognitive dysfunction likely intensify caregiver burden; currently, however, there are limited data qualifying this association. Previous studies for evaluating caregiver burden in this patient group have been limited by use of non-specific survey instruments [11, 14, 22]. However, caregiver burden, for those caring for people with malignant glioma, has been shown to be significantly higher than for those caring for patients with other cancers [18]. A survey instrument is under development to evaluate burden and QoL in caregivers of patients with glioblastoma and cognitive dysfunction [3, 4]. This survey was used in the current study to evaluate associations between patient clinical characteristics and caregiver burden/QoL.

## Materials and methods

### Study design

This study used a mixed-methods design, including parallel and cross-sectional comparisons with both a prospective component and a retrospective component. A national cohort of caregiver–patient dyads across 7 academic cancer centers in the United States were included in the study from April 2018 through June 2019. The 7 centers included 4 Oncology Research Information Exchange Network (ORIEN) [27] member sites (Huntsman Cancer Institute, Moffitt Cancer Center, the James Cancer Hospital at the Ohio State University, and University of Colorado Cancer Center) and 3 academic sites with expertise in glioblastoma (Duke University; University of California, Los Angeles; University of California, San Francisco). Each center obtained local scientific and institutional review board approval, recruited study participants, and securely transmitted de-identified data to the University of Utah for analysis. Caregivers and patients gave written informed consent to participate in the study; patients had given broad consent previously to have access to their medical records, which were used in this study.

Caregivers were surveyed once each with the new glioblastoma-specific caregiver burden instrument [3, 4] and the Caregiver Quality of Life Index-Cancer (CQOL-C) questionnaire [38]. The new glioblastoma-specific caregiver burden instrument is currently being developed, and is designed to assess the impact of each of the 7 domains of caregiver burden included in the survey scored from 1 to 10 [3, 4]. This survey has not yet been validated. The survey explored caregiver general burden, including emotional health, change in cognitive function of loved one with glioblastoma, information needs, caregiving responsibilities, caregiver’s health needs, and caregiver’s work and finances. These domains were evaluated with a focus on the impact of patients’ cognitive dysfunction on caregiver burden. CQOL-C is a validated, 35-item questionnaire that evaluates facets of a caregiver’s well-being and determines a final score on a scale of 0 to 100; a higher score represents better QoL [38]. The CQOL-C questionnaire has previously been shown to correlate with the instrument used in this analysis [3, 4]. At the time that a caregiver completed the surveys, clinical data of their respective patient currently living with glioblastoma were abstracted from medical records via an automated data pull of discrete variables and/or review of clinical notes facilitated by a standardized case report form built in REDCap® [28].

### Eligibility criteria

Eligible caregiver participants were aged ≥ 18 years who self-identified as the primary caregiver of an individual living with glioblastoma; acknowledged fifth-grade or higher literacy proficiency in English at screening; indicated perceiving cognitive dysfunction in the patient they cared for; and had not participated in the pilot study. Patients with glioblastoma were eligible if they were alive at the time their caregiver enrolled in the study; were aged ≥ 18 years when diagnosed with glioblastoma; had confirmed diagnosis by two *International Classification of Diseases (ICD)-9* (191.7, 191.8, 191.9) or *ICD-10* codes (V10.85, 71.9), physician documentation, or *ICD-O-3* or pathology-confirmed diagnosis; and had two or more healthcare encounters with one encounter at least 30 days from index date (date of diagnosis of glioblastoma).

### Data collection

At each center, a research coordinator approached caregivers accompanying their patient to a clinic visit. Caregivers who consented to participate completed surveys electronically via Qualtrics® or on paper. Caregivers who started the survey electronically in Qualtrics completed their survey using a unique link that allowed them to access their incomplete survey electronically. Caregivers who started the survey in paper format completed the survey on paper and arranged with the study coordinator to return the survey in person or by mail.

### Statistical analysis

Caregiver demographics and patient demographic and clinical characteristics were summarized using descriptive statistics. Patients were additionally summarized by subgroups by tumor MGMT methylation status and Karnofsky performance status scores. Patients were stratified by tumor MGMT status as it is a known prognostic factor that is prevalent in approximately 50% of people with glioblastoma. This allowed the assessment of any difference in methylated/unmethylated MGMT status on patient characteristics. Comparisons of patient characteristics between patient subgroups were evaluated using tests of association, including Chi square, Fisher’s exact, Wilcoxon rank-sum, analysis of variance, and Student’s *t* test, as appropriate. The Karnofsky performance status rubric defines patients with Karnofsky performance status ≥ 80 as able to carry on normal activity with no special care needed. Patients with a score of 80 have some signs or symptoms of disease, and those with 90 and 100 exhibit minor and no signs of disease, respectively. Predictive models for caregiver burden (defined as the impact of each of the 7 domains of caregiver burden included in the survey) were built using a backward elimination variable selection method. Given that the purpose of this model is to identify risk factors for increased caregiver burden in the glioblastoma population, variable selection was conducted based on a two-part approach. In step 1, a comprehensive list of demographic, clinical, and caregiver-reported variables was evaluated in a univariable model for association with caregiver burden. In step 2, a backward elimination process was conducted for all variables that showed an unadjusted association with caregiver burden in step 1 (i.e., variables that met a strict criterion for significance [*p* < 0.05]). To decrease the risk of false-positive associations, variables that had a *p* value > 0.05 were removed from the multivariable model one-by-one until the model included only significant risk factors for caregiver burden. STATA version 14.2 was used for statistical analysis.

## Results

### Patient demographics

The final cohort included 167 dyads recruited from 7 academic cancer sites between November 2018 and June 2019. These 167 dyads represent a subgroup of 185 dyads originally recruited, for which results on caregiver perceptions of cognitive dysfunction have previously been reported [3, 4]. There were 18 caregivers who completed the survey for which consent by the patient they cared for was not obtained. The mean (± standard deviation [SD]) age of patients at diagnosis was 57 (± 12) years, and the majority of patients were white (87%) and male (62%) (Table 1). Ten percent of patients had multifocal lesions and 60% had baseline Karnofsky performance status scores of 80–100. No significant differences in demographic features were observed for patients with methylated MGMT (mMGMT) versus unmethylated MGMT (uMGMT) tumor status. Overall, 23 patients (14%) had an *IDH1* mutation–positive tumor. More patients with mMGMT versus uMGMT tumor status had *IDH1* mutation–positive tumors (*n* = 13 vs 5, *p* = 0.05) (Table 1). Five patients (3%) had tumors with concurrent alterations in *TERT* and *IDH1*. This study did not distinguish between primary and secondary glioblastoma. At the time of survey completion, 44% of caregivers reported that patients were receiving treatment for initial glioblastoma diagnosis, 38% reported that patients were receiving treatment for glioblastoma recurrence, 15% reported that patients were not receiving treatment, and 2% reported that patients were receiving hospice/symptom management, respectively (patient treatment status was unknown for 1%).

**Table 1:** Patient characteristics at baseline

Characteristic	Total (*N* = 167)	Methylated MGMT (*n* = 70)	Unmethylated MGMT (*n* = 67)	Unknown MGMT (*n* = 30)	*p* value^a^
Age at index					
Age, mean (SD)	57 (12.4)	60 (10.9)	56 (12.8)	55 (14.4)	0.09^d^
Age ≥ 65 years, *n* (%)	42 (31)	21 (30)	21 (31)	8 (27)	0.87^b^
Male, *n* (%)	103 (62)	40 (57)	46 (69)	17 (57)	0.16^b^
Race, *n* (%)					0.73^c^
American Indian or Alaska Native	2 (1)	1 (1)	1 (1)	0 (0)	
Asian	1 (1)	0 (0)	0 (0)	1 (3)	
Black	8 (5)	2 (3)	5 (7)	1 (3)	
White	146 (87)	63 (90)	57 (85)	26 (87)	
Hispanic	6 (4)	2 (3)	3 (4)	1 (3)	
Other	4 (2)	1 (1)	2 (3)	1 (3)	
Location of lesion, *n* (%)					0.23^b^
Right side, frontal	30 (18)	9 (13)	18 (27)	3 (10)	
Left side, frontal	26 (16)	7 (10)	12 (18)	7 (23)	
Right side, parietal	15 (9)	8 (11)	5 (7)	2 (7)	
Left side, parietal	14 (8)	7 (10)	5 (7)	2 (7)	
Right side, temporal	27 (16)	17 (24)	9 (13)	1 (3)	
Left side, temporal	18 (11)	8 (11)	7 (10)	3 (10)	
Other	37 (22)	14 (20)	11 (16)	12 (40)	
Karnofsky performance status score at baseline, *n* (%)					0.36^c^
100	12 (7)	2 (3)	9 (13)	1 (3)	
90	50 (30)	22 (31)	20 (30)	8 (27)	
80	38 (23)	15 (21)	13 (19)	10 (33)	
70	12 (7)	5 (7)	5 (7)	2 (7)	
≤ 60	5 (3)	1 (1)	1 (1)	3 (10)	
Unknown	49 (30)	25 (36)	19 (28)	6 (20)	
Genomic alterations, *n* (%)^e^					
*IDH1*	23 (14)	13 (19)	5 (7)	5 (17)	0.05^b^
Charlson Comorbidity Index score at time of diagnosis, mean (SD)	0.94 (2.1)	0.99 (2.1)	0.78 (2.1)	1.2 (2.5)	0.56^d^
Multifocal, *n* (%)	16 (10)	6 (9)	6 (9)	4 (13)	0.94^b^
Other cancer, *n* (%)^f^	34 (20)	16 (23)	11 (16)	7 (23)	0.34^b^
Family history of brain cancer or other cancers, *n* (%)					0.98^b^
Yes	71 (43)	26 (37)	26 (39)	19 (63)	
No	64 (38)	30 (43)	28 (42)	6 (20)	
Unknown	32 (19)	14 (20)	13 (19)	5 (17)	

*MGMT* O6-methylguanine-DNA methyltransferase promoter, *mMGMT* methylated MGMT, *uMGMT* unmethylated MGMT, *SD* standard deviation

^a^mMGMT vs uMGMT. ^b^Chi square test. ^c^Fisher’s exact test. ^d^Student’s *t* test. ^e^Other genomic alterations were evaluated but did not show any significant differences. These included *BRAF, EGFR, EGFR VIII, PDGFR, TERT, TERT* and *IDH1*, and other. ^f^Other cancers included any other malignancy, solid tumor, or blood malignancy that the patient had previously or at time of the survey completion, which were not glioblastoma

### Caregiver demographics

Caregivers were predominantly white (82%) and female (63%), and had a mean (± SD) age of 58 (± 12) years. The majority had attended college/graduate school (77%) and 36% reported household incomes > $100,000. Before glioblastoma diagnosis, 59% of caregivers were employed; at the time of enrollment, 44% of caregivers were employed. Many were patients’ spouses (84%). Forty-seven percent of caregivers had provided care for 6 to 23 months.

### Treatment patterns

TMZ monotherapy was the most common first-line systemic treatment (89% of patients). Few patients received combination systemic therapy as initial treatment (Supplementary Table 1).

### Cognitive dysfunction

**Fig. 1:**
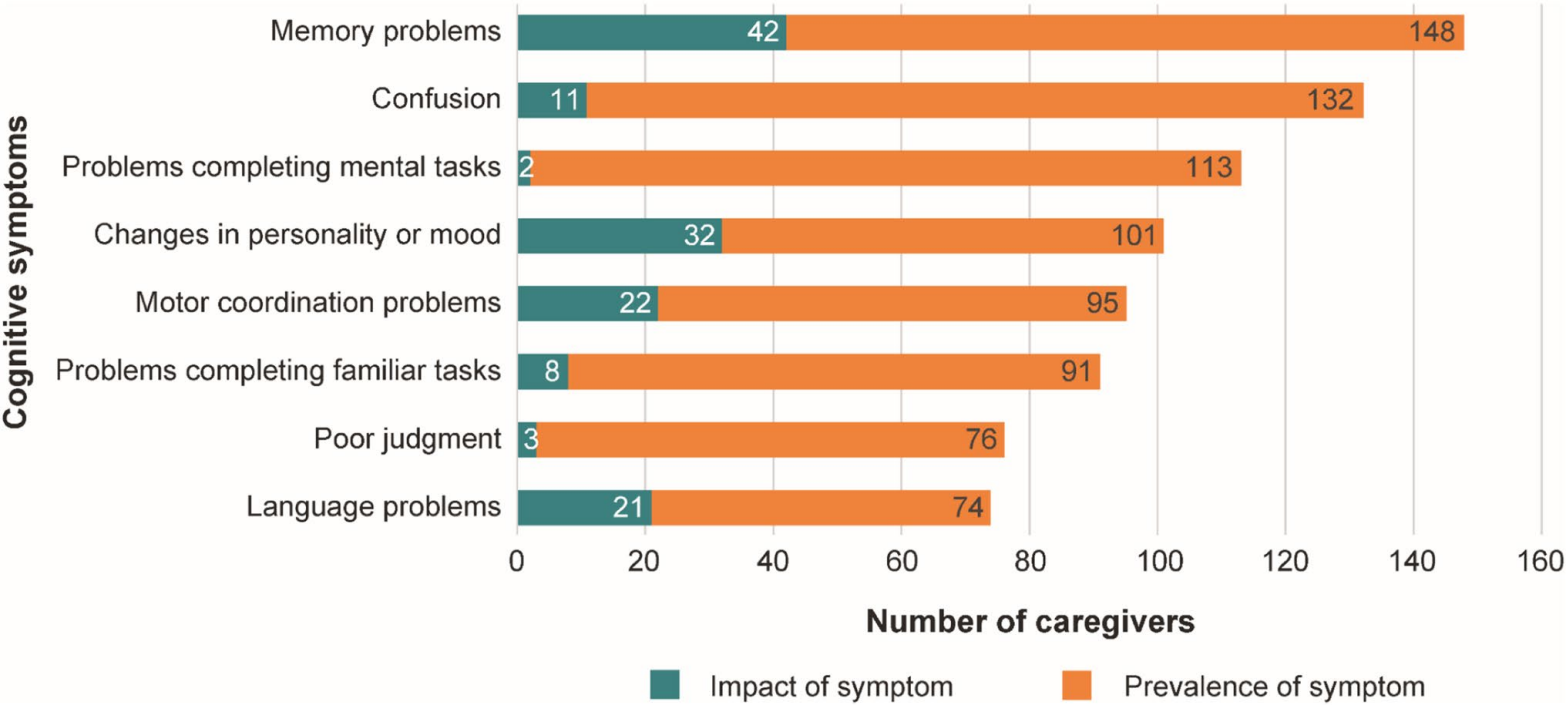
Prevalence and impact of specific cognitive symptoms

When asked about the impact of cognitive dysfunction symptoms, caregivers ranked memory problems, changes in personality/mood, and language problems in their patient as the most impactful to them daily (Fig. 1).

Caregivers ranked memory problems, confusion, and problems completing mental tasks as the 3 most common cognitive dysfunction symptoms observed in patients in the last 14 days. More caregivers of patients with mMGMT tumor status, rather than uMGMT tumor status, reported their patient having problems completing familiar tasks (67% vs 45%, *p* = 0.049) and problems completing mental tasks (82% vs 61%, *p* = 0.04) (Table 2).

**Table 2:** Association of caregivers’ perception of cognitive dysfunction and clinical characteristics

Clinical characteristic	Cognitive dysfunction symptom							
	Memory problems	Language problems	Confusion	Problems completing familiar tasks	Problems completing mental tasks	Changes in personality or mood	Poor judgment	Body function or motor coordination problems
MGMT methylation status								
Methylated MGMT (*n* = 70)	65 (94)	29 (45)	57 (84)	44 (67)	55 (82)	42 (63)	31 (47)	34 (52)
Unmethylated MGMT (*n* = 67)	55 (83)	31 (47)	51 (77)	30 (45)	40 (61)	42 (65)	30 (46)	38 (57)
Unknown MGMT (*n* = 30)	28 (93)	14 (48)	24 (83)	17 (59)	18 (64)	17 (59)	15 (50)	23 (77)
*p* value	0.21^b^	0.99^b^	0.61^a^	**0.049**^b^	**0.04**^b^	0.90^b^	0.31^b^	0.12^b^
Karnofsky performance status score								
90–100 (*n* = 62)	53 (88)	25 (42)	46 (77)	29 (48)	35 (57)	35 (58)	19 (32)	31 (52)
≤ 80 (*n* = 55)	51 (93)	23 (43)	46 (85)	34 (63)	41 (77)	34 (64)	32 (59)	35 (65)
Unknown (*n* = 50)	44 (88)	26 (55)	40 (82)	28 (61)	37 (79)	32 (67)	25 (53)	29 (60)
*p* value	0.68^b^	0.37^b^	0.51^a^	0.28^b^	**0.03**^b^	0.67^b^	**0.01**^b^	0.53^b^
Surgery at diagnosis								
Yes (*n* = 153)	135 (90)	66 (46)	119 (80)	82 (57)	103 (73)	91 (63)	67 (47)	84 (58)
No (*n* = 14)	13 (93)	8 (57)	13 (93)	9 (64)	10 (77)	10 (77)	9 (64)	11 (79)
*p* value	> 0.99^b^	0.43^a^	0.47^b^	0.58^a^	> 0.99^b^	0.38^b^	0.20^a^	0.14^a^
Recurrence								
No (*n* = 67)	60 (90)	25 (40)	49 (77)	35 (56)	46 (72)	39 (61)	34 (53)	38 (58)
Yes (*n* = 100)	88 (90)	49 (51)	83 (84)	56 (57)	67 (69)	62 (64)	42 (43)	57 (59)
*p* value	0.88^b^	0.25^b^	0.25^a^	> 0.99^b^	0.26^b^	0.66^b^	**0.03**^b^	0.41^b^
Number of lesions								
Multifocal (*n* = 16)	14 (88)	7 (44)	12 (75)	10 (63)	12 (80)	10 (67)	7 (47)	11 (73)
Unifocal (*n* = 151)	134 (90)	67 (47)	120 (82)	81 (56)	101 (69)	91 (62)	69 (47)	84 (57)
*p* value	0.66^b^	0.78^a^	0.51^b^	0.65^a^	0.76^b^	> 0.99^b^	0.91^a^	0.27^b^
Location of lesion								
Frontal (*n* = 56)	45 (82)	23 (43)	49 (82)	33 (60)	34 (67)	35 (66)	27 (50)	37 (67)
Not frontal (*n* = 111)	103 (94)	51 (50)	86 (80)	58 (56)	79 (76)	66 (63)	49 (47)	58 (56)
*p* value	**0.01**^a^	0.41^a^	0.79^a^	0.61^a^	0.22^a^	0.69^a^	0.73^a^	0.18^a^
Side of lesion								
Left (*n* = 89)	78 (89)	55 (63)	70 (80)	46 (54)	60 (72)	51 (60)	39 (46)	47 (55)
Right (*n* = 78)	70 (92)	19 (27)	62 (82)	45 (61)	53 (74)	50 (68)	37 (51)	48 (67)
*p* value	0.46^a^	** < 0.01**^a^	0.86^a^	0.40^a^	0.85^a^	0.27^a^	0.55^a^	0.12^a^
*TERT/IDH1* status								
*TERT*+ and *IDH1*+ (*n* = 5)	3 (60)	2 (50)	3 (75)	3 (75)	3 (75)	4 (80)	2 (50)	2 (50)
Not *TERT*+ and *IDH1*+ (*n* = 162)	145 (91)	72 (47)	129 (81)	88 (57)	110 (73)	97 (63)	74 (48)	93 (60)
*p* value	0.08^b^	> 0.99^b^	0.57^b^	0.64^b^	> 0.99^b^	0.65^b^	> 0.99^b^	> 0.99^b^

Significant *p* values (< 0.05) are in bold

*MGMT* O6-methylguanine-DNA methyltransferase promoter

^a^Chi square test. ^b^Fisher’s exact test

Analyses were carried out for MGMT/*IDH1* status and *IDH1* status; however, as *n* values were very low, these data were not included in the table

All data are *n* (%), unless otherwise stated; missing responses were excluded from the analysis and the denominator is based on the number of caregivers that answered each question

### Caregiver tasks and time

Caregiving tasks, including “mobility and transportation,” “meal preparation,” “bathroom needs,” “medication organization/administration,” “other household tasks (e.g., cleaning, grocery shopping),” and “other caregiving activities,” were evaluated for association with patient clinical characteristics. More caregivers of patients with low versus high Karnofsky performance status reported performing tasks related to “mobility and transportation” (87% for Karnofsky performance status ≤ 80 vs 68% for Karnofsky performance status 90–100, *p* < 0.01) and “bathroom needs” (44% for Karnofsky performance status ≤ 80 vs 21% for Karnofsky performance status 90–100, *p* < 0.01). Similarly, more caregivers of patients with a lesion involving the frontal lobe indicated performing tasks related to “bathroom needs” compared with caregivers of patients with a lesion not in the frontal lobe (43% vs 28%, *p* = 0.05). More caregivers of patients with concurrent mMGMT and *IDH1* mutation–positive tumor status reported performing tasks related to “medication organization/administration” compared with patients with concurrent uMGMT and *IDH1* mutant tumor status (77% vs 20%, *p* = 0.047). No associations were seen between caregiving tasks and surgery at initial diagnosis, number or site of lesions, *TERT* and *IDH1* mutation status, *IDH1* mutation status alone, or length of time as caregiver (results not shown). Patients who received partial resections were significantly more likely to require assistance using the bathroom (*p* < 0.01). No other associations were found between type of surgery and caregiver tasks.

Caregiving time and patient clinical characteristics were also evaluated. Caregivers were asked, “On average, how many hours each week do you spend directly providing care as a result of the glioblastoma diagnosis? Examples include preparing meals, grooming, lifting, and administering medication.” Time was categorized as 1 to 3, 4 to 6, 7 to 9, 10 to 12, and > 12 h weekly. More caregivers of patients with lower baseline Karnofsky performance status compared with higher Karnofsky performance status devoted > 12 h weekly for providing care (58% for Karnofsky performance status ≤ 80 vs 29% for Karnofsky performance status 90–100, *p* < 0.01). No associations were seen between caregiving time and glioblastoma recurrence, surgery at initial diagnosis, extent of surgery, number/location/side of lesion, or tumor genomics (*TERT* and *IDH1* mutation status, MGMT and *IDH1* mutation status, *IDH1* mutation status alone) (results not shown).

### Caregiver QoL

The overall median CQOL-C score was 81.5 (interquartile range, 68–98). Median CQOL-C score was higher among caregivers of patients with a tumor involving the frontal lobe compared with caregivers of patients with non-frontal lobe lesions (89 vs 78.5, *p* = 0.04). No significant associations were seen between caregiver QoL and glioblastoma recurrence, baseline Karnofsky performance status, surgery at diagnosis, number or site of lesions, tumor MGMT status, *TERT* and *IDH1* mutation status, MGMT methylation and *IDH1* mutation status, or *IDH1* mutation status alone.

### Predictive factors for caregiver burden

Different domains of caregiver burden were examined with univariable and multivariable models, including clinical and nonclinical variables. Multivariable analysis showed that patient changes in personality/mood versus no changes (*p* = 0.01), patient confusion (yes vs no, *p* < 0.01), and caregiver task of “medication administration” (yes vs no, *p* = 0.02) were independent predictors of poorer emotional health of the caregiver (Table 3). Patient sex (male vs female, *p* < 0.01), time commitment > 12 h weekly versus < 12 h weekly (*p* = 0.01), patient confusion (yes vs no, *p* < 0.01), and “transportation” (yes vs no, *p* = 0.02) were independent contributors to predicting poorer caregiver social health. Similarly, patient sex (male vs female; *p* < 0.01), time commitment > 12 h weekly versus < 12 h weekly (*p* = 0.01), and patient confusion (yes vs no, *p* < 0.01) were independent contributors to predicting poorer caregiver general health (Table 3). Multivariable analysis showed that independent predictors of caregiver inability to work included patient confusion (yes vs no, *p* = 0.01), patient poor judgment (yes vs no, *p* = 0.01), and providing medication (yes vs no, *p* < 0.01) (Table 4). Independent contributors to poor confidence in finances, as assessed by the caregiver, included patient sex (male vs female, *p* = 0.03), older caregiver age (*p* < 0.01), patient confusion (yes vs no, *p* < 0.01), and “bathroom needs” (yes vs no, *p* < 0.01). Lastly, patient confusion and poor judgment (yes vs no, both *p* < 0.01) were significantly associated with poor caregiver overall QoL (Table 4).

**Table 3:** Multivariable models for caregiver health

		Emotional health			Social health			General health		
Variable	Comparator	*n*	Regression coefficient (95% CI)	*p *value	*n*	Regression coefficient (95% CI)	*p *value	*n*	Regression coefficient (95% CI)	*p *value
Patient sex	Male vs female	161			158	1.71 (0.76–2.65)	** < 0.01**	158	1.70 (0.77–2.62)	** < 0.01**
Time commitment	> 12 vs < 12 h	151			150	1.32 (0.29–2.34)	**0.01**	149	1.22 (0.28–2.16)	**0.01**
Patient symptoms in last 14 days										
Changes in personality or mood	Yes vs no	154	1.36 (0.30–2.42)	**0.01**	151			151		
Confusion	Yes vs no	159	2.46 (1.14–3.78)	** < 0.01**	156	2.66 (1.43–3.88)	** < 0.01**	157	2.55 (1.34–3.76)	** < 0.01**
Transportation	Yes vs no	161			158	1.69 (0.30–3.08)	**0.02**	158		
Medication administration	Yes vs no	161	1.54 (0.26–2.82)	**0.02**	158			158		

Significant *p* values (< 0.05) are in bold

*CI* confidence interval

**Table 4:** Multivariable models for caregiver ability to work, confidence in finances, and overall quality of life

		Ability to work			Confidence in finances			Overall quality of life			
Variable	Comparator	Number	Regression coefficient (95% CI)	*p* value	Number	Regression coefficient (95% CI)	*p* value	Number		Regression coefficient (95% CI)	*p* value
Patient sex	Male vs female	142			150	1.09 (0.09–2.08)	**0.03**	158			
Caregiver age	Continuous (increasing)	142			150	−0.08 (−0.12–−0.04)	** < 0.01**	158			
Patient symptoms in last 14 days											
Confusion	Yes vs no	140	2.09 (0.50–3.68)	**0.01**	148	1.97 (0.69–3.24)	** < 0.01**	156		2.28 (1.00–3.56)	** < 0.01**
Poor judgment	Yes vs no	136	1.62 (0.40–2.84)	**0.01**	145			151		1.42 (0.42–2.42)	** < 0.01**
Caregiving responsibilities											
Bathroom needs	Yes vs no	142			150	1.64 (0.59–2.69)	** < 0.01**	158			
Medication administration	Yes vs no	142	2.39 (0.84–3.94)	** < 0.01**	150			158			

Significant *p* values (< 0.05) are in bold

*CI* confidence interval

## Discussion

This study used a novel survey explicitly designed to measure caregiver burden in those caring for patients with glioblastoma. Patient clinical factors affecting caregiver QoL were also assessed. Patients in this study were representative of the general glioblastoma patient population, with a predominance of males (1.6 × females) and initial presentation with a solitary lesion [31, 35]. Median age at diagnosis (59.2 years) was younger than in other reports (64 years) [35].

More than 90% of patients received guideline-supported first-line treatment. The majority of patients in this study had a good baseline performance status, with 85% of patients with a known Karnofsky performance status scoring > 80. Distributions of tumor MGMT status and *EGFR*, *TERT*, and *IDH1* genomic alterations observed in the current study are consistent with those reported in the current literature [1, 5, 7, 10]. Methylation of MGMT in glioblastoma tumors occurs in approximately half of patients and is considered an independent prognostic marker for survival, and is predictive of response to TMZ [16, 21, 34, 39]. Among patients in this study with known tumor MGMT status, 51% had mMGMT.

Characteristics of caregivers of patients with glioblastoma in this study were mostly similar to those of other cancer caregivers; most caregivers were female and related to the patient, but they were older than the average cancer caregiver by 5 years and had achieved a higher level of education [23, 30]. Patient clinical characteristics influenced manifestations of cognitive dysfunction and caregiver experience in this study. Problems completing familiar tasks and problems completing mental tasks were associated with mMGMT tumor status. Lower baseline Karnofsky performance status was associated with difficulties completing mental tasks and poor judgment in the current study. These symptoms, such as memory loss, exemplify executive functions typically controlled by the brain’s frontal lobe [19]. However, in the current study, significantly more caregivers of patients with a lesion outside the frontal lobe reported that their patient had memory problems. In addition, memory problems were considered the most common and most impactful cognitive dysfunction symptom for caregivers on a daily basis. These results illustrate the complexity of neuro-oncological manifestations of glioblastomas as well as the caregiver experience. 

Analysis of caregiving tasks and caregiver time demonstrated that significantly more time was required for “mobility and transportation” and “bathroom needs” for patients with lower baseline Karnofsky performance status. This finding is consistent with augmented caregiving typically needed for individuals with reduced functioning, even when comparing small differences in Karnofsky performance status scores. In this study, Karnofsky performance status scores were recorded at initial diagnosis. We did not evaluate if this decline was driven by disease alone and/or treatment toxicity, and the Karnofsky performance status score of the patient at the time of the caregiver survey was not recorded.

In this study, caregiver QoL was unaffected by the patient’s clinical characteristics except for the location of lesion, with a higher CQOL-C score significantly associated with tumor involving the frontal lobe. Since cognitive functions are primarily controlled by the frontal lobe, surgical excision of lesions from this area may produce more obvious improvements and higher perceived benefit, which could have affected the caregiver’s QoL.

The predictive models examining facets of caregivers’ health, ability to work, confidence in finances, and overall QoL illustrate the complexity of the caregiver experience. A common theme was the impact of patient confusion, which was an independent contributor to all these domains, although source of patient confusion was not evaluated. Nevertheless, future caregiver interventions should address this symptom. Caregivers could benefit from education about the development or worsening of confusion and receiving advice on practical techniques to manage situations where it plays a large role, especially if there is a safety concern. They also should be urged to initiate advanced care planning before confusion prohibits patient decision-making [13]. Caregivers could be encouraged to track details about their patient’s confusion to help determine its cause. For example, increased confusion may be seen after treatment with chemotherapy, radiation therapy, or steroids [9]. This information would be helpful toward development of future treatments for glioblastoma, or new modalities or modification of current therapies, that would enable effective control of disease with minimal neurological adverse effects.

A key strength of this study is that caregiver burden/QoL was evaluated against respective patient clinical characteristics across a national cohort [3, 4]. Collaborating sites in this cohort are specialized cancer centers with expertise in glioblastoma. Inclusion of living patients with glioblastoma also strengthened this study by minimizing caregiver recall bias. However, this study has several limitations. Recall bias may exist despite attempts to minimize this by enrolling only caregivers of living patients and asking about cognitive dysfunction symptoms in the last 14 days. In addition, “better-faring patient” bias similar to survival bias may exist. Multiple questions in the glioblastoma survey used a Likert scale [3, 4]; although this is a convenient and easy-to-understand measure, it assumes an even interval between measuring points. As previously noted, the new glioblastoma-specific caregiver burden instrument employed in this study is currently being developed and is not yet validated; however, its use in this study serves as an important resource in the ongoing validation process of this novel instrument. Results from this study come from a one-time survey administration and do not reflect the dynamic process of caregiving [24]. Some caregivers did not answer all the survey questions, leading to some missing data. However, questions had a random distribution of missing data with the majority of questions having missing responses from < 10% of caregivers. In those questions missing > 10% of responses, questions were skipped by some caregivers as some questions were not applicable to the whole group, i.e., a question about work skipped by a caregiver who does not work. Patient data were collected from medical records and are thus subject to the inherent biases of retrospective studies, such as missing or incomplete data. Due to the lack of previous research on caregiving burden in the glioblastoma population, the variable selection for these models was largely exploratory. Since the *p* values assume that each variable was pre-specified [33], the models created should be verified in future studies. The caregivers included in the survey were predominantly white and well educated and reported a very good income due to the location of the study centers involved in the survey development. As a result, we acknowledge that selection bias may be present. Finally, caregiver evaluations, including evaluations of the patients’ cognitive function, in this study were subjective, which may have led to potential bias in answers to survey questions.

## Conclusion

The results of this study have significant implications for clinical practice. Previously, no instrument had been available that specifically focused on caregiver burden, for caregivers of patients with glioblastoma. This study demonstrated differences in the impact of patient baseline function on caregivers, the prevalence of patient confusion on multiple areas of caregiver life and burden, and the overall idea that not all glioblastoma caregiver–patient dyads are alike. These could lead to close collaboration among the patient, the caregiver, and the oncology multidisciplinary team to support caregivers who could benefit from interventions tailored to their individual circumstances. Early identification of specific caregiver domains affected by caregiving could be the first step in triggering an intervention by the multidisciplinary team. New caregivers overwhelmed by their new role could be encouraged to attend supportive care sessions that review practical strategies to help with daily activities of caregiving (nurse), including medication organization (pharmacist), transportation assistance (social work), creating a care network (social work), and optimizing home health services (nurse), all toward avoiding caregiver burnout. Veteran caregivers are more likely to experience caregiver burnout, and many caregivers develop mental health problems, including anxiety and depression [15, 25]; caregivers for patients with brain cancer experience lower levels of mental health than those in other cancers [2]. As such, caregivers would benefit from supportive care sessions aimed at reviewing self-care, normalization of feelings, and finding an appropriate patient–caregiver time balance, as well as support from a psychologist or a psychiatrist for assistance with mental health conditions. From a patient’s perspective, shared decision-making can provide a central role for the patient in the decision-making process through open communication, which could also help the caregiver provide optimal care to the patient [6].

This study used a new instrument that is being developed to assess how caregiver burden is affected by the patient’s clinical factors. Re-evaluation of this survey over the course of the disease would allow future research to investigate the dynamic process of caregiving. The variables included in the models as well as the reliability of the results should be verified in future studies to improve the measurement of burden among caregivers of patients with glioblastoma. In addition, evaluating caregiver QoL by geography, such as community cancer centers in rural areas, may highlight disparities in caregiver burden.

## Supplementary Information

Below is the link to the electronic supplementary material.

Supplementary file1 (DOCX 17 KB)

## Data Availability

Not applicable.

## Code availability

STATA V15.1 was used for all analyses.
