# Correction to: Caregiver burden by treatment and clinical characteristics of patients with glioblastoma

**DOI:** 10.1007/s00520-021-06691-y

**Published:** 2021-11-18

**Authors:** Trang H. Au, Connor Willis, Maija Reblin, Katherine B. Peters, Phioanh Leia Nghiemphu, Jennie W. Taylor, Howard Colman, Adam L. Cohen, D. Ryan Ormond, Arnab Chakravarti, Nicole Willmarth, Jyothi Menon, Junjie Ma, Hillevi Bauer, Alexandre H. Watanabe, Cornelia M. Ulrich, Prianka Singh, Alexander Marshall, Beata Korytowsky, David Stenehjem, Diana Brixner

**Affiliations:** 1 Department of Pharmacotherapy, College of Pharmacy, University of Utah, Salt Lake City, UT USA; 2 Department of Health Outcomes and Behavior, Moffitt Cancer Center, Tampa, FL USA; 3 The Preston Robert Tisch Brain Tumor Center, Duke University Medical Center, Durham, NC USA; 4 Department of Neurology, University of California, Los Angeles, Los Angeles, CA USA; 5 Departments of Neurology and Neurological Surgery, University of California, San Francisco, San Francisco, CA USA; 6 Department of Neurosurgery, Huntsman Cancer Institute, University of Utah, Salt Lake City, UT USA; 7 Division of Oncology, Department of Internal Medicine, Huntsman Cancer Institute, University of Utah, Salt Lake City, UT USA; 8 Department of Neurosurgery, University of Colorado School of Medicine, Aurora, CO USA; 9 Department of Radiation Oncology, The Ohio State University College of Medicine, Columbus, OH USA; 10 American Brain Tumor Association, Chicago, IL USA; 11 Huntsman Cancer Institute and Department of Population Health Sciences, University of Utah, Salt Lake City, UT USA; 12 Bristol Myers Squibb, Princeton, NJ USA; 13 Department of Pharmacy Practice and Pharmaceutical Sciences, College of Pharmacy, University of Minnesota, Duluth, MN USA

**Correction to: Supportive Care in Cancer**

10.1007/s00520-021-06514-0

The article " Caregiver burden by treatment and clinical characteristics of patients with glioblastoma", written by Au, T.H., Willis, C., Reblin, M., Peters, K.B., Nghiemphu, P.L., Taylor, J.W., Colman, H., Cohen, A.L., Ormond, D.R., Chakravarti, A., Willmarth, N., Menon, J., Ma, J., Bauer, H., Watanabe, A.H., Ulrich, C.M., Singh, P., Marshall, A., Korytowsky, B., Stenehjem, D., Brixner, D., was originally published electronically on the publisher’s internet portal on 12 September 2021 without open access. With the author(s)’ decision to opt for Open Choice the copyright of the article changed on 16 November 2021 to © The Author(s) 2021 and the article is forthwith distributed under a Creative Commons Attribution 4.0 International License, which permits use, sharing, adaptation, distribution and reproduction in any medium or format, as long as you give appropriate credit to the original author(s) and the source, provide a link to the Creative Commons licence, and indicate if changes were made. The images or other third party material in this article are included in the article’s Creative Commons licence, unless indicated otherwise in a credit line to the material. If material is not included in the article’s Creative Commons licence and your intended use is not permitted by statutory regulation or exceeds the permitted use, you will need to obtain permission directly from the copyright holder. To view a copy of this licence, visit

http://creativecommons.org/licenses/by/4.0.

The original article has been corrected.

