# Supplementary material for: Caregiver burden by treatment and clinical characteristics of patients with glioblastoma
Source: Support Care Cancer. 2021 Sep 11;30(2):1365–75. doi: 10.1007/s00520-021-06514-0 (PMC8727395; doi:10.1007/s00520-021-06514-0)
Supplement: Supplementary file 1 — Supplementary file1 (DOCX 17 KB) [file 520_2021_6514_MOESM1_ESM.docx]

**Supplementary Table 1**. Treatment patterns at initial diagnosis

| **Initial treatment, *n* (%)** | **Total (*N* = 167)** | **Methylated MGMT (*n* = 70)** | **Unmethylated MGMT (*n* = 67)** | **Unknown MGMT^a^ (*n* = 30)** |
| --- | --- | --- | --- | --- |
| **Surgery** | 153 (92) | 65 (93) | 63 (94) | 25 (83) |
| **Radiation** | 160 (96) | 68 (97) | 64 (96) | 28 (93) |
| **Radiation plus TMZ** | 148 (89) | 63 (90) | 58 (87) | 27 (90) |
| **Systemic treatment** | 163 (98) | 67 (96) | 67 (100) | 29 (97) |
| **Only chemotherapy agents, *n*** | 122 (73) | 54 (77) | 47 (70) | 21 (70) |
| TMZ monotherapy | 109/122 (89) | 48/54 (89) | 42/47 (89) | 20/21 (95) |
| TMZ + gliadel | 5/122 (4) | 2/54 (4) | 3/47 (6) | 0 (0) |
| TMZ + lomustine | 3/122 (2) | 2/54 (4) | 1/47 (2) | 0 (0) |
| TMZ + other chemo | 1/122 (1) | 1/54 (2) | 0 (0) | 0 (0) |
| TMZ + gliadel + lomustine | 1/122 (1) | 0 (0) | 0 (0) | 1/21 (5) |
| Lomustine monotherapy | 1/122 (1) | 0 (0) | 1/47 (2) | 0 (0) |
| Lomustine + procarbazine + vincristine | 1/122 (1) | 1/54 (2) | 0 (0) | 0 (0) |
| **Chemotherapy + targeted therapy** | 13 (8) | 3 (4) | 5 (7) | 5 (17) |
| TMZ + other targeted | 9/13 (69) | 1/3 (33) | 4 (80) | 4 (80) |
| TMZ + bevacizumab | 2/13 (15) | 2/3 (67) | 0 (0) | 0 (0) |
| TMZ + lomustine + other targeted | 1/13 (8) | 0 (0) | 1/4 (20) | 0 (0) |
| TMZ + other chemo + other targeted | 1/13 (8) | 0 (0) | 0 (0) | 1/4 (20) |
| **Chemotherapy + immunotherapy** | 2 (1) | 1 (1) | 1 (1) | 0 (0) |
| TMZ + other chemo + pembrolizumab | 2/2 (100) | 1/2 (100) | 1/1 (100) | 0 (0) |
| **Chemotherapy + clinical trial drug** | 15 (9) | 6 (9) | 7 (10) | 2 (7) |
| TMZ + clinical trial | 14/15 (93) | 5/6 (83) | 7/7 (100) | 2/2 (100) |
| TMZ + other chemo + clinical trial | 1/15 (7) | 1/6 (17) | 0 (0) | 0 (0) |
| **Targeted therapy monotherapy** | 3 (2) | 0 (0) | 2 (3) | 1 (3) |
| Bevacizumab | 0 (0) | 0 (0) | 0 (0) | 0 (0) |
| Other targeted therapy | 3/3 (100) | 0 (0) | 2/2 (100) | 1/1 (100) |
| **Immunotherapy monotherapy** | 0 (0) | 0 (0) | 0 (0) | 0 (0) |
| **Only clinical trial** | 8 (5) | 3 (4) | 5 (7) | 0 (0) |
| **No systemic therapy/unknown** | 4 (2) | 3 (4) | 0 (0) | 1 (3) |

*MGMT* O^6^-methylguanine-DNA methyltransferase promoter, *TMZ* temozolomide.

^a^Includes patients with unavailable MGMT status. Results not shown here.
